# Immunotherapy targeting the *Streptococcus pyogenes* M protein or streptolysin O to treat or prevent influenza A superinfection

**DOI:** 10.1371/journal.pone.0235139

**Published:** 2020-06-23

**Authors:** Andrea L. Herrera, Christopher Van Hove, Mary Hanson, James B. Dale, Rodney K. Tweten, Victor C. Huber, Diego Diel, Michael S. Chaussee

**Affiliations:** 1 Division of Basic Biomedical Sciences, The Sanford School of Medicine of the University of South Dakota, Vermillion, SD, United States of America; 2 Department of Medicine, Division of Infectious Diseases, University of Tennessee Health Science Center, Memphis, TN, United States of America; 3 Department of Microbiology and Immunology, University of Oklahoma Health Sciences Center, Oklahoma City, OK, United States of America; 4 Department of Veterinary and Biomedical Sciences, South Dakota State University, Brookings, SD, United States of America; Oregon Health & Science University, UNITED STATES

## Abstract

Viral infections complicated by a bacterial infection are typically referred to as coinfections or superinfections. *Streptococcus pyogenes*, the group A streptococcus (GAS), is not the most common bacteria associated with influenza A virus (IAV) superinfections but did cause significant mortality during the 2009 influenza pandemic even though all isolates are susceptible to penicillin. One approach to improve the outcome of these infections is to use passive immunization targeting GAS. To test this idea, we assessed the efficacy of passive immunotherapy using antisera against either the streptococcal M protein or streptolysin O (SLO) in a murine model of IAV-GAS superinfection. Prophylactic treatment of mice with antiserum to either SLO or the M protein decreased morbidity compared to mice treated with non-immune sera; however, neither significantly decreased mortality. Therapeutic use of antisera to SLO decreased morbidity compared to mice treated with non-immune sera but neither antisera significantly reduced mortality. Overall, the results suggest that further development of antibodies targeting the M protein or SLO may be a useful adjunct in the treatment of invasive GAS diseases, including IAV-GAS superinfections, which may be particularly important during influenza pandemics.

## Introduction

Infection with influenza A virus (IAV) creates a permissive environment for secondary bacterial infections (often referred to as superinfections), which significantly increases the morbidity and mortality associated with both IAV epidemics and pandemics [1]. *Streptococcus pyogenes* (the group A Streptococcus, GAS) typically causes pharyngitis but also can cause more serious invasive GAS infections (iGAS) such as bacteremia, toxic shock syndrome, necrotizing fasciitis, and IAV superinfections. An analysis of lung biopsies from the 1918 influenza pandemic revealed that *Streptococcus pneumoniae* and GAS were the most frequently observed bacteria in the lungs and contributed to approximately 90% of the estimated 50 million deaths attributed to influenza [2].

IAV increases host susceptibility to secondary bacterial infections by a variety of mechanisms. Most research related to GAS has focused on the characterization of viral-induced changes affecting GAS adherence and internalization [3–7]. Consistent with these studies, murine models of IAV-GAS superinfection show that IAV infection significantly enhances the virulence of GAS [7–9]. In addition, active vaccination of mice against IAV provides significant protection against GAS secondary infections indicating a specific role for the virus in enhancing host susceptibility to superinfection [10, 11]. Finally, epidemiological studies support the idea that several viruses, and particularly influenza, increase the incidence of iGAS diseases including pneumonia [3, 12].

Many antibiotics, including penicillin, are effective against GAS. Nonetheless, iGAS diseases are associated with a surprisingly high fatality rate. For example, during the 2009 IAV pandemic, 7 out of 10 patients in California with a laboratory confirmed IAV-GAS superinfection died despite being treated with antibiotics and anti-viral agents. The median age at the time of death was 37 years [13]. In a separate study conducted between December 2010 and January 2011, 14 of 19 patients with an iGAS disease also had an IAV infection; ten died, even though at least nine received antibiotics effective against GAS *ex vivo* [14]. Finally, it is estimated that while only 12% of iGAS infections involve the lower respiratory tract [15], 38% are fatal [16]. Thus, while GAS remains susceptible to many antibiotics and influenza vaccines and anti-viral agents are widely used, the mortality of IAV-GAS superinfections is significant [3].

Currently there are no vaccines available for GAS; however, vaccination with the surface-localized M protein elicits protective opsonic antibodies [17–20] and experimental M protein-based vaccines have been used in animal studies [21] and human clinical trials [22, 23]. In addition, we previously showed that active vaccination targeting the M protein confers 100% protection against mortality by using a murine model of IAV-GAS superinfection [24].

Another potential GAS vaccine target is the secreted cholesterol-dependent cytolysin (CDC) streptolysin O (SLO). SLO contributes to virulence [25] and orthologues are encoded in the genomes of a wide range of bacteria [26] including *Streptococcus pneumoniae* (pneumolysin; PLY), which has historically been the most frequent cause of IAV superinfections [27]. Passive immunotherapy with anti-PLY antibodies protects mice against *S*. *pneumoniae* bacteremia [28], indicating that the cytolytic CDC toxins may be good candidates for passive immunotherapy targeting bacterial pathogens associated with IAV superinfections.

While the development of an effective active vaccine against GAS is an ideal outcome, we were interested in assessing the efficacy of using passively administered antibodies to prevent or treat IAV-GAS superinfections. In the current study, we evaluated the prophylactic and therapeutic use of antisera targeting either the M protein or SLO in a murine model of IAV-GAS superinfection.

## Materials and methods

### Bacterial and viral isolates and culture conditions

*S*. *pyogenes* strain MGAS315 (serotype M3) was obtained from ATCC and grown statically with Todd-Hewitt broth, or agar plates (BD Biosciences, San Jose, CA) supplemented with 0.2% yeast extract (THY) at 37°C in 5% CO_2_. To prepare stocks to inoculate mice, GAS was grown overnight with THY agar, colonies were inoculated into pre-warmed THY medium, grown to the mid-exponential phase of growth (*A*_600_  =  0.5), and diluted in sterile phosphate-buffered saline (PBS; pH 7.4). The number of viable GAS was determined by dilution plating.

Influenza virus A/Puerto Rico/8/34-H1N1 (PR8), was propagated for 72 hours at 35°C in the allantoic cavities of 10-day-old embryonated chicken eggs [24, 29]. Viral RNA was extracted, reverse transcribed, and whole genome sequence analyses were used to confirm the genetic makeup of the viral preparation. Further characterization of the virus included calculating the median tissue culture infectious dose (TCID_50_) in Madin-Darby Canine Kidney (MDCK) cells.

### Polyclonal antiserum

A recombinant hexavalent M protein vaccine containing protective M protein peptides from GAS serotypes M24, M5, M6, M19, M1, and M3 was generated by amplifying and ligating the 5’ end of each *emm* gene together into a pQE-30 vector [30]. The SLO toxoid was created by mutating amino acids (Thr and Leu) in the domain that comprises the cholesterol binding motif [31]. The hexavalent M protein or SLO toxoid were used to generate polyclonal antibodies in rabbits, as described previously [32]. Briefly, 1 mL of each purified recombinant protein (1.6 mg M protein or 2.5 mg SLO toxoid) was mixed 1:1 with the adjuvant Montanide ISA 50 (Seppic Inc; Fairfield, New Jersey). Adult 12-week old rabbits were kept in individual cages with food and water *ad libitum* throughout the study. Each rabbit was immunized with either antigen, which was administered by three 0.5 mL subcutaneous injections (day 0, 14, and 28) and one 0.5 mL intramuscular injection (day 0). The rabbits were euthanized by exsanguination 42 days after the initial injection.

### Superinfection of mice and passive immunization

All experiments were conducted in conformity with the recommendations in the Guide for the Care and Use of Laboratory Animals of the National Institutes of Health and according to the guidelines of the local Institutional Animal Care and Use Committee of the University of South Dakota (protocol number 10-06-18-21E). Drs. Victor Huber and Ruth Bakker (D.V.M.) provided all animal care and handling training. Female (6 to 8-week-old) BALB/c mice were purchased from Jackson Laboratories (Indianapolis, IN) and housed in groups of four with 24-hour access to food and water. The IAV-GAS superinfection model was previously described and we showed that IAV (PR8) persists in the lungs of mice up to day 9 after infection; which overlaps with GAS infection on day 7[33]. Mice were lightly anesthetized with 2.5% isoflurane and IAV or GAS was given intranasally (100 μl total volume). Intranasal administration was performed by pipetting small droplets every 2 to 3 seconds (approximately 5–10 μl) onto the outer edge of each nostril (alternating) where it was inhaled until 100 μl was delivered. For IAV and GAS the LD_50_ was determined by the method of Reed and Muench [34]. We used 0.1 LD_50_ as a sub-lethal dose, which was 10^0.75^ TCID_50_ of IAV and 10^6^ CFUs of GAS [11, 24]. For superinfections, mice were inoculated with IAV on day 0 and GAS on day 7. Animal health and behavior was monitored at least three times a day over the 21 day course of the experiment. Body weight was recorded daily. Endpoint criteria included extreme clinical signs of infection (huddling, hunched posture, ruffled fur, tachypnea), severe hypothermia as indicated by a temperature of 34°C (~4.5°C below normal), and weight loss equal to or greater than 20% of starting weight. Mice with one or more of these symptoms were immediately euthanized and the infection was considered lethal. The total number of mice used in the study was 138. Of these, 61 were euthanized and 77 succumbed to infection.

Polyclonal serum (125 μL) was administered either intraperitoneally (i.p.) using a 25-gauge needle, which was inserted at approximately 45° into the side of the abdominal wall, or administered intranasally (100 μL).

### Antisera neutralization of SLO hemolytic activity

The capacity of SLO antisera to neutralize SLO cytolytic activity was determined as previously described [35]. Briefly, 100 μg of purified SLO toxin was serially diluted 2-fold with PBS (50 μl total volume) in a 96-well flat bottom plate. Then, 50 μl of SLO antiserum, non-immune serum, or PBS, was added and the plates were incubated at 37°C for 60 minutes. Next, 50 μl of 5% rabbit erythrocytes (Innovative Research; Novi, MI) was added to each well and incubated for an additional 30 minutes at 37°C. Intact erythrocytes were removed by centrifugation at 4,000 rpm for 5 minutes. Hemoglobin present in the supernatants was measured by determining the *A*_540_. The effective concentration of SLO required for 50% lysis (EC_50_) was determined with GraphPad Prism.

### GAS quantification in mouse tissues

Mouse tissue samples were collected 24 hours after GAS inoculation (day 8). Blood was collected from the submandibular vein of each animal and immediately after they were euthanized by CO_2_ inhalation the lungs and spleens were removed and homogenized in sterile PBS. GAS were enumerated by dilution plating with THY or blood agar plates as previously described [11].

### Enzyme-Linked Immunosorbent Assays (ELISA)

ELISA was done as previously described [21]. Briefly, 96-well plates were coated with 5 μg/mL of recombinant M protein or the SLO toxoid diluted with 0.1 M sodium carbonate (pH 9.8). Plates were blocked with 1% BSA, washed with PBS containing 0.05% Tween 20 (PBS-T), and serial dilutions of sera were added to the wells and incubated for 2 hours at 37°C. Plates were washed again with PBS-T, and HRP-conjugated goat anti-rabbit IgG (H+L) (Sigma, St. Louis, MO) was added to each well. After washing, HRP was detected using One-Step-TMB Turbo substrate (Thermo Scientific, Rockford, IL). The OD was measured at 450 nm using a Biotek EL808 plate reader (Biotek, Winooski, VT). End-point titers were defined as the reciprocal serum dilution corresponding with the last well demonstrating an OD_450_ of 0.1 in the titration curve. In some experiments, intact MGAS315 was immobilized to the ELISA plate as the antigen.

### Bacterial killing in whole blood

Antibody-mediated killing of GAS was measured with whole mouse blood (Biochemed Services; Winchester VA). Stocks of GAS were diluted in PBS to 10^4^ CFUs and mixed with 100 μg of IgG (diluted in PBS) and incubated at room temperature for 15 minutes to allow antibodies to bind GAS. Then, 200 μL of whole mouse blood was added and incubated for 30 minutes at 37°C and 5% CO_2_. Viable GAS were enumerated by dilution plating on THY agar. Cytochalasin D inhibits actin polymerization, and is a general inhibitor of phagocytosis. In some experiments cytochalasin D (final concentration, 10 μM; Sigma-Aldrich, St. Louis, MO) was added to whole blood 30 minutes prior to the addition of GAS to inhibit phagocytosis.

### IgG purification

The IgG fraction of antibodies was isolated from polyclonal rabbit sera using a 1 mL Melon Gel column, as per manufacturer’s instructions (Thermo Fisher Scientific; Rockford, IL). All purified IgG was immediately buffer exchanged to a final concentration of 1 mg/mL in PBS using 40 kDa Zeba spin columns (Thermo Fisher Scientific). IgG fractionation was verified by SDS-PAGE and concentrations were determined by absorbance at 280 nm using an extinction coefficient (E280 0.1%) of 1.4.

### Statistical analysis

Data were analyzed using GraphPad Prism software (GraphPad Software, Inc., La Jolla, CA), and values were accepted as significant if *P* < 0.05. Statistical analyses included a one-way or two-way analysis of variance (ANOVA) with a Tukey’s multiple comparisons post-hoc test, Kaplan Meier survival analysis, or a Student’s t-test, as appropriate. All figures were created using GraphPad Prism software.

## Results

### Characterization of antisera to SLO and the M protein

**Fig 1:**
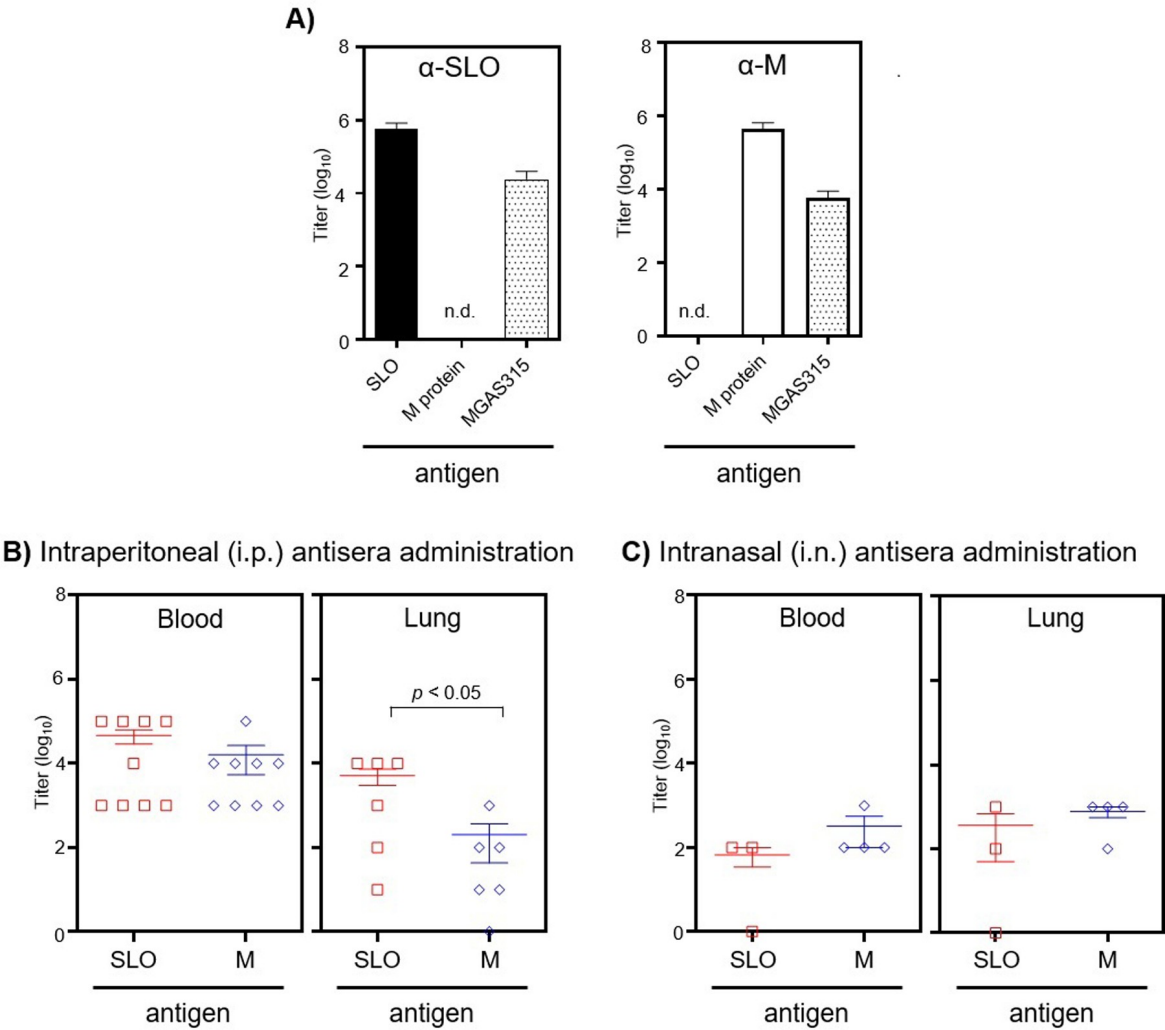
Antisera against SLO or the M protein bound to purified antigen and to immobilized GAS strain MGAS315. **(A)**. The titers were determined with ELISA. Data shown are the means ± sem from 4 to 5 independent experiments. n.d. indicates antibody binding was not detected; α refers to antisera. ELISA was also used to determine the titers of antibodies specific to SLO (open square) or the M protein (open diamond) in the blood and lungs of mice 24 hours after **B)** i.p. or **C**) i.n administration of antisera. The means ± sem from 3 to 9 mice are indicated. Statistical significance was determined with the Student's t-test.

Rabbits were injected with either a recombinant hexavalent M-protein vaccine, which included an amino acid sequence known to elicit antibodies specific to the serotype M3 protein [30] or with an SLO toxoid [26]. The titers of antigen-specific antibodies against the SLO or the M protein vaccines were determined by using an enzyme-linked immunosorbent assay (ELISA) and were 600,000 and 400,000 respectively (Fig 1). Antisera to SLO and to the M protein also contained antibodies that bound to an immobilized serotype M3 strain MGAS315 (antibody titers of 22,000 and 6,000 respectively), (Fig 1). The results indicated that the epitopes of the target proteins were exposed and accessible to the antibodies. Non-immune serum, which in subsequent experiments was used as a negative control, also contained a low level of antibodies that bound to immobilized MGAS315 (antibody titer 1,000).

### Determination of antibody titers to SLO and the M protein in the blood and lungs of mice following intraperitoneal (i.p.) or intranasal (i.n.) administration of antisera

Prior to assessing the efficacy of using passively administered antisera to curtail disease, it was of interest to measure the titers of antibodies in mice following either i.p. or i.n. administration of antisera. Antisera to either SLO or the M protein was given separately by either i.p. (125 μL) or i.n. (100 μL) inoculation. After 24 h, the titers of antigen-specific antibodies in the blood and lungs of mice were determined with ELISA (Fig 1B and 1C). Following i.p. administration, the antibody titers to SLO (50,000) and the M protein (20,000) were similar in the blood (*p* > 0.05); however, the average antibody titer to SLO (5,000) was greater in the lungs compared to the titer of M protein-specific antibodies (<1,000; *p* < 0.05; Fig 1B). There was significantly greater variation in antibody titers among lung samples compared to blood samples following i.p. injection (SLO *F*_8,5_ = 95, *p* < 0.0005 and M protein *F*_8,5_ = 6560, *p* < 0.0005; Fig 1B). Following i.n. administration of antisera, the antibody titers to SLO and the M protein in both the blood and lungs were similar and below 1,000 (Fig 1C).

### Growth inhibition of GAS in murine blood in the presence of antibodies to the M protein and antibody-mediated neutralization of SLO cytolytic activity

**Fig 2:**
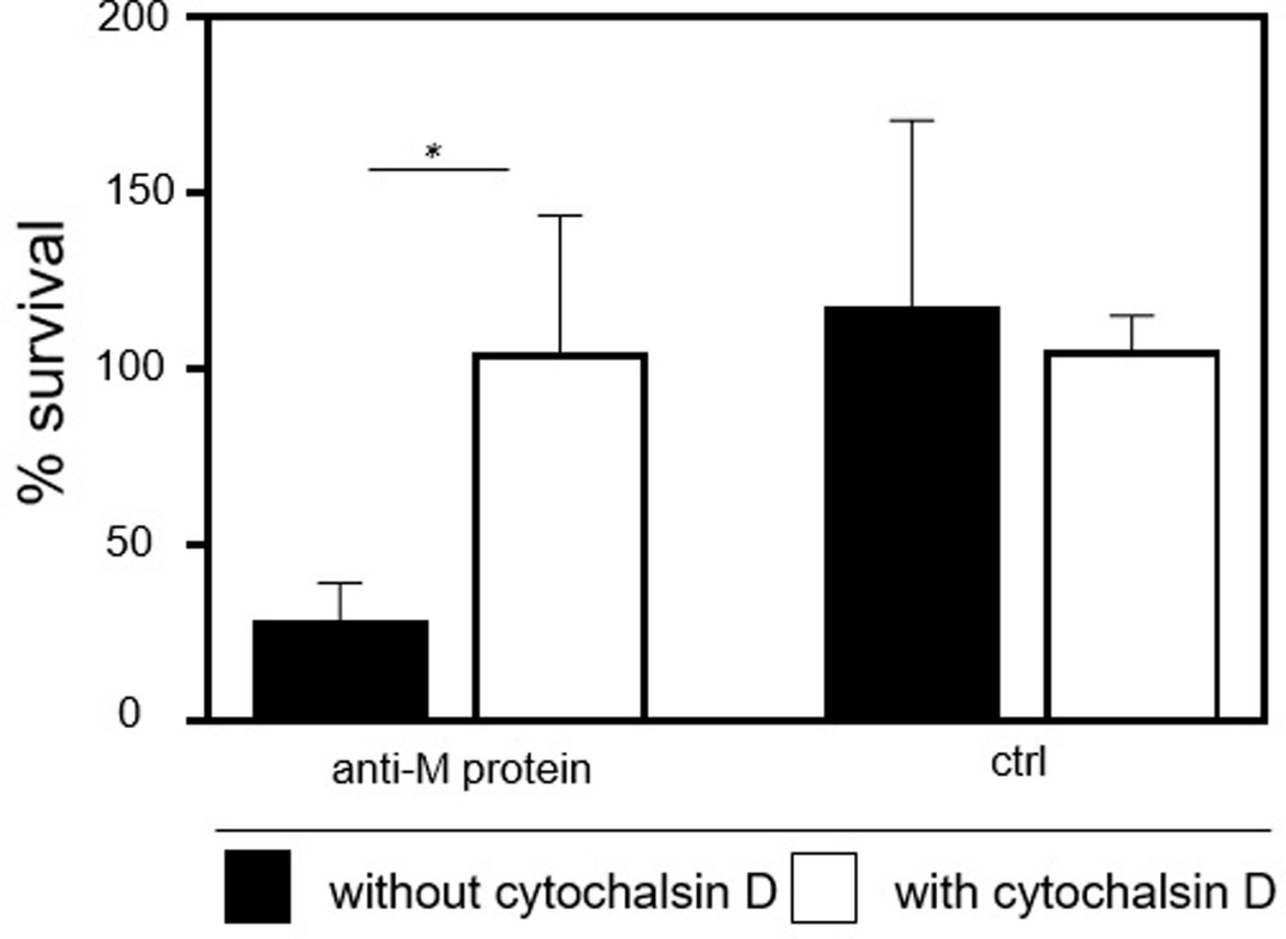
Antibodies to the M protein inhibit growth of GAS in blood. Growth and viability of GAS in murine blood. GAS was pre-incubated for 30 min. with IgG to the M protein or non-immune IgG (ctrl) prior to suspension in murine blood that contained, or not, cytochalasin D. After an additional 30 min., viable bacteria were enumerated by dilution plating. The results shown are the means ± sem from 4 independent experiments. Statistical significance was determined with the Student's t-test.

We next determined if the IgG fraction of the antisera to the M protein could inhibit the growth of GAS in the presence of phagocytes and complement. For this, GAS was incubated for 30 minutes with the IgG fraction of antisera raised against the M protein; controls included non-immune rabbit IgG. The bacteria were then suspended with murine blood for 30 minutes at 37°C and viable bacteria were enumerated by dilution plating. As an additional control, cytochalasin D was used to inhibit actin polymerization and phagocytosis. Compared to non-immune IgG, the addition of antibodies to the M protein decreased the number of viable GAS by 90%; however, the difference was not statistically significant (Fig 2). The addition of cytochalasin D inhibited the killing of GAS incubated with antibodies to the M protein (*p* < 0.05; Fig 2); however, the addition of cytochalasin D did not inhibit the killing of GAS incubated with non-immune IgG. The results suggested the antibodies to the M protein enhanced phagocytosis and killing of GAS in murine blood.

To determine if the antisera to SLO contained antibodies that neutralized SLO cytolytic activity, we measured SLO-mediated hemolysis in the presence of antiserum to SLO, non-immune serum, or PBS. The SLO-specific antisera inhibited hemolytic activity compared to controls (*p* < 0.0001; Table 1) indicating the sera contained neutralizing antibodies.

**Table 1:** Hemolytic activity of SLO in the presence of serum.

Serum	EC_50_ (nM)^a^	Neutralization of [SLO] compared to control (*p*-value)^b^
Control^c^	5.1	-
Non-immune	2.6	0.5 fold (*p* = 0.999)
Anti–SLO	1,900	373-fold (*p* < 0.0001)

^*a*^ The mean effective SLO concentration for 50% lysis of rabbit erythrocytes (EC_50_) as determined from 3 independent experiments.

^*b*^ A one-way analysis of variance (ANOVA) with a Tukey’s multiple comparisons post-hoc test was used to determine significance of differences in [SLO] between sera and the control (PBS).

^*c*^ PBS.

### Determination of the abundance of GAS in the lungs, spleens, and blood of IAV-GAS superinfected mice treated prophylactically with antisera to SLO or the M protein

**Fig 3:**
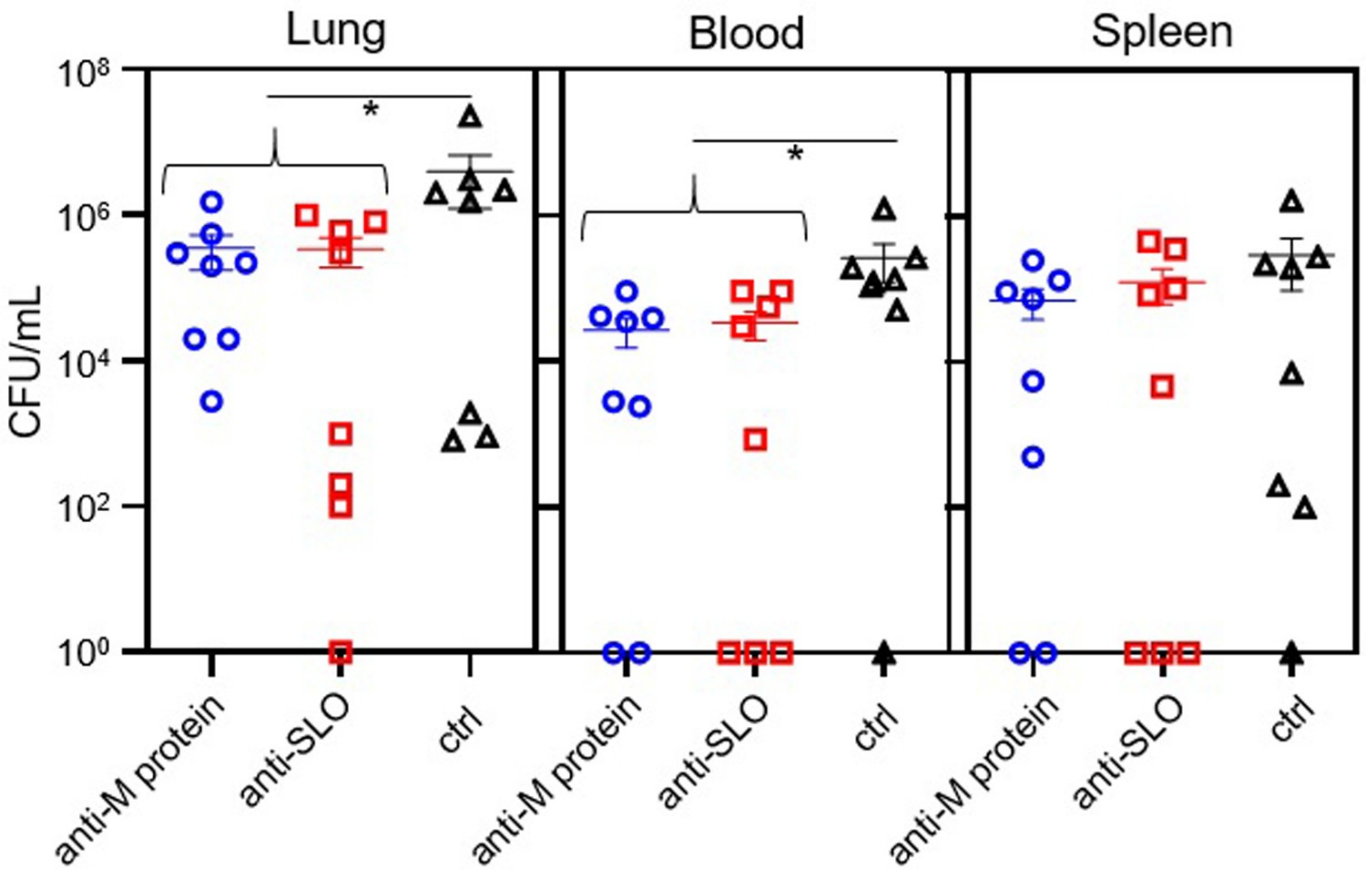
Quantification of GAS in the lungs, blood, and spleens of mice treated prophylactically with antisera. Mice were infected i.n. with 0.1 LD_50_ IAV on day 0. On day 7, mice were treated with antisera specific to either the M protein or SLO, or non-immune sera (ctrl) and superinfected 6 h later with GAS (0.1 LD_50_). Mice were euthanized 24 h later and the number of GAS present in blood, lungs, and spleens was determined by dilution plating. Differences between the individual groups were not statistically significant (*p* > 0.05); however, the difference in the number of bacteria in the lungs and blood of mice treated with either antisera to SLO or the M protein was lower compared to those treated with non-immune sera (*p* < 0.05; *). Statistical significance was determined with the Student's t-test.

To determine if prophylactic immunotherapy can decrease the bacterial burden among IAV-GAS superinfected mice, we quantified GAS in the lungs, blood, and spleen of superinfected mice. To do so, mice were infected i.n. with a sublethal dose of IAV (0.1 LD_50_). Seven days later they were given antisera to either SLO or the M protein, or non-immune serum. Six hours later, mice were inoculated i.n. with a sublethal dose of GAS (0.1 LD_50_). After 24 h, the mice were euthanized and the number of viable bacteria in the lungs, blood, and spleen was determined by dilution plating. The differences among individual groups treated with antisera compared to controls (e.g. antisera to M protein versus control sera) were not statistically significant (*p* > 0.05; Fig 3); however, when results were analyzed by comparing the combination of mice treated with either antisera, there were fewer bacteria in the lungs and blood (*p* < 0.05) of mice compared to mice treated with non-immune sera.

### Determination of the morbidity and mortality of superinfected mice treated prophylactically with antisera to SLO or the M protein

**Fig 4:**
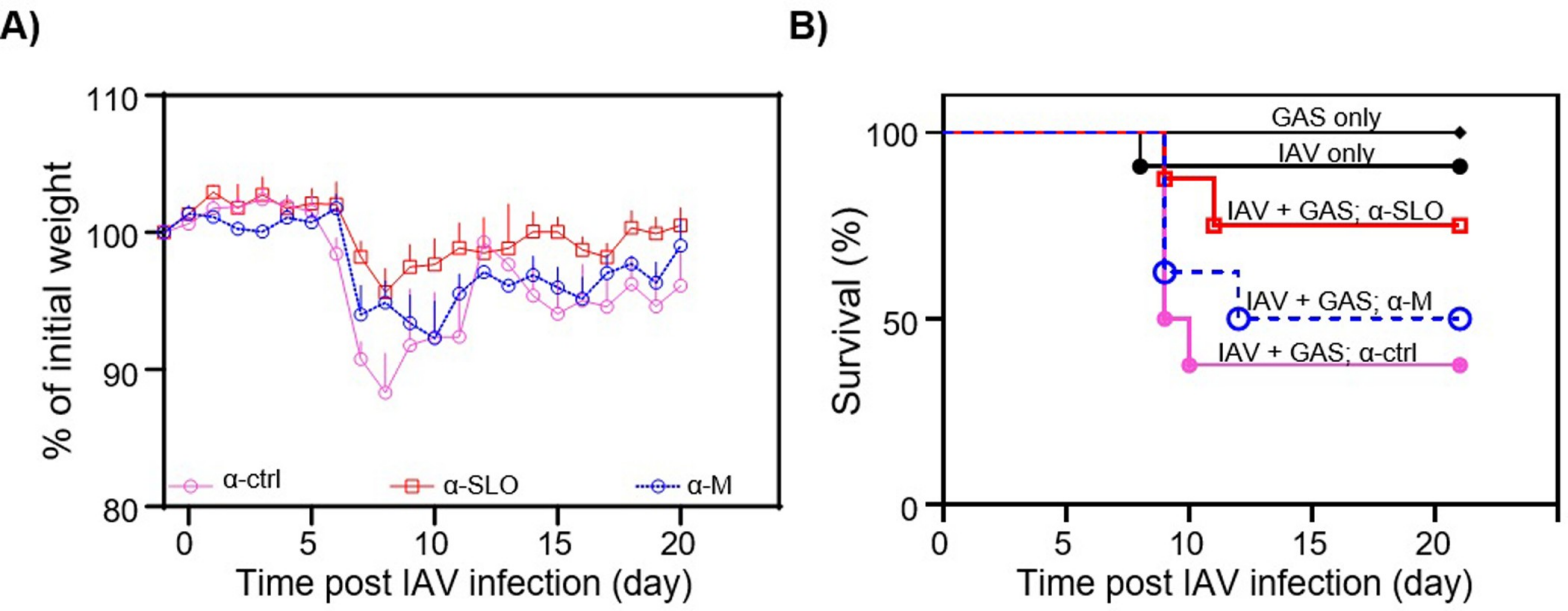
Determination of the effect of prophylactic administration of antisera to SLO (α-SLO) or the M protein (α-M) on morbidity (weight loss) and mortality of IAV-GAS superinfected mice. **A)** Treatment with α-SLO and α-M decreased morbidity compared to groups of mice treated with non-immune sera (*p* < 0.001) **B)** The mortality associated with IAV-GAS superinfection was slightly reduced following administration of either α-SLO or α-M compared to treatment with non-immune sera (*p* > 0.05). Control groups also included untreated mice inoculated with IAV alone (day 0) or GAS alone (day 7). The area under the curve analysis (days 8–15) was completed prior to an unpaired Student’s *t*-test to compare the differences in morbidity. A Kaplan–Meier survival analysis was used to compare the differences in mortality.

To determine if prophylactic use of antisera to SLO or the M protein could decrease either the morbidity or mortality associated with IAV-GAS superinfection, groups of mice were first inoculated with a sublethal dose of IAV (0.1 LD_50_) on day 0. On day 7 mice were given antisera to either SLO or the M protein, or non-immune rabbit sera. Six hours later, mice were inoculated with a sublethal dose of GAS (0.1 LD_50_). Additional untreated groups of mice included those infected with a sublethal dose (0.1 LD_50_) of either IAV or GAS alone to control for the synergistic effects of viral-bacterial superinfection. Prophylaxis with antisera specific to SLO and to the M protein significantly decreased morbidity compared to mice receiving non-immune sera (Fig 4A; *p* < 0.001). Prophylaxis with antisera to either SLO or the M protein also increased survival by 37.5% and 12.5%, respectively compared to mice receiving non-immune sera (*p* > 0.05; Fig 4B).

### Determination of the abundance of GAS in the lungs, spleens, and blood of IAV-GAS superinfected mice treated with antisera to SLO or the M protein

**Fig 5:**
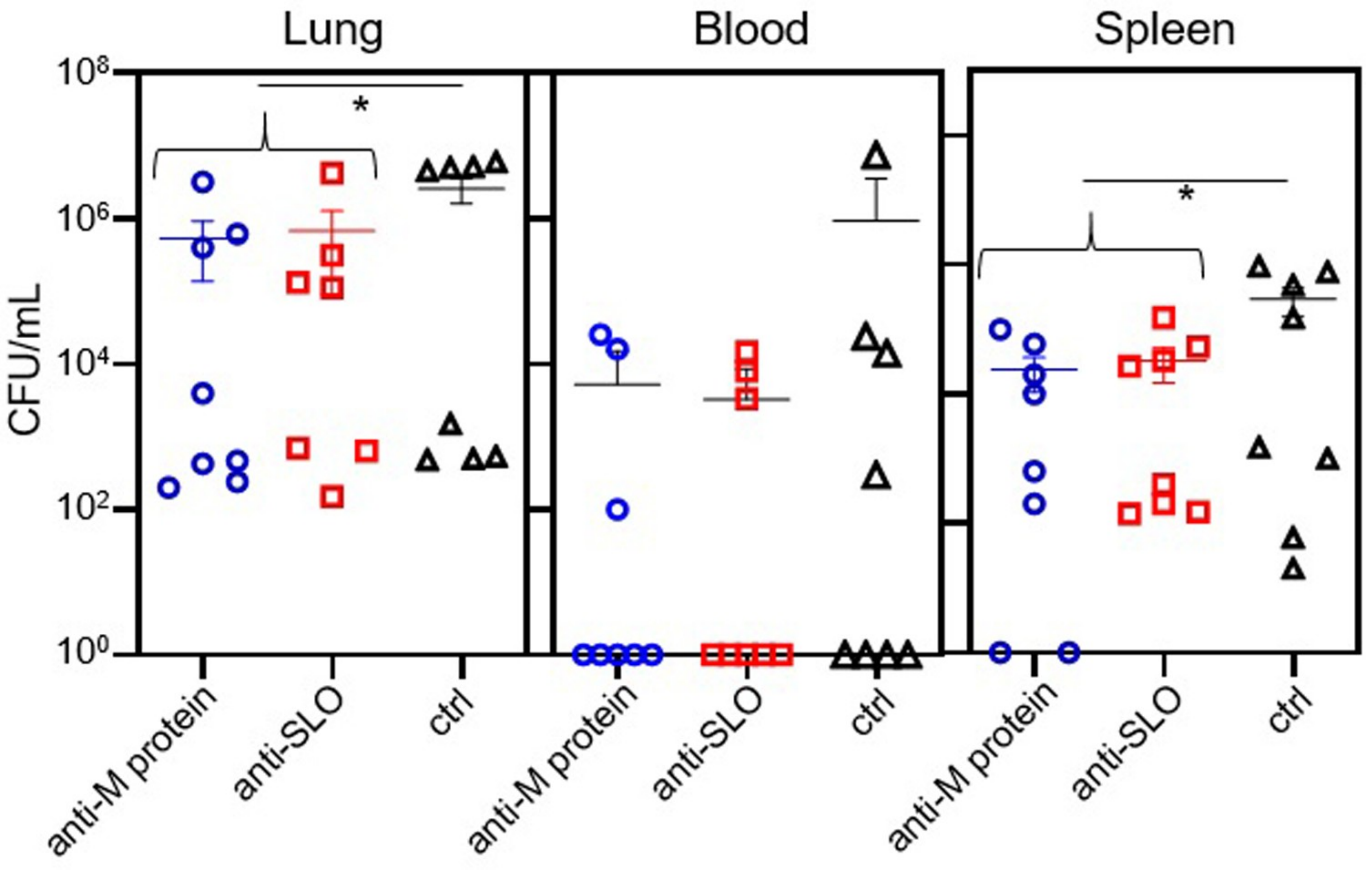
Quantification of GAS in the lungs, blood, and spleens of mice treated with antisera 2 h after superinfection. Mice were infected i.n. with 0.1 LD_50_ IAV on day 0 and GAS on day 7 followed 2 h later by treatment with antisera to the M protein, SLO, or non-immune sera as a control (ctrl). Mice were euthanized 24 h later and the number of GAS present in blood, lungs, and spleens was determined by dilution plating. Differences between the individual groups were not statistically significant (*p* > 0.05); however, there were fewer GAS in the lungs and spleen of mice treated with either antisera to SLO or the M protein compared to those treated with non-immune sera (*p* < 0.05; *). Statistical significance was determined with the Student's t-test.

To determine if immunotherapy administered after the establishment of an IAV-GAS superinfection could enhance the clearance of GAS from mice, we quantified GAS in the lungs, blood, and spleen of superinfected mice treated with antisera to either SLO or the M protein. The differences between each individual group treated with immune sera compared to mice treated with non-immune sera (e.g. antisera to M protein versus control sera) were not statistically significant (*p* > 0.05; Fig 5); however, when we compared mice treated with antisera to either SLO or the M protein (as a combined group) the mice treated with GAS-specific antisera had fewer GAS in the lungs and spleens compared to mice treated with non-immune sera (*p* < 0.05; Fig 5).

### Determination of the morbidity and mortality of superinfected mice treated with antisera to SLO or the M protein

**Fig 6:**
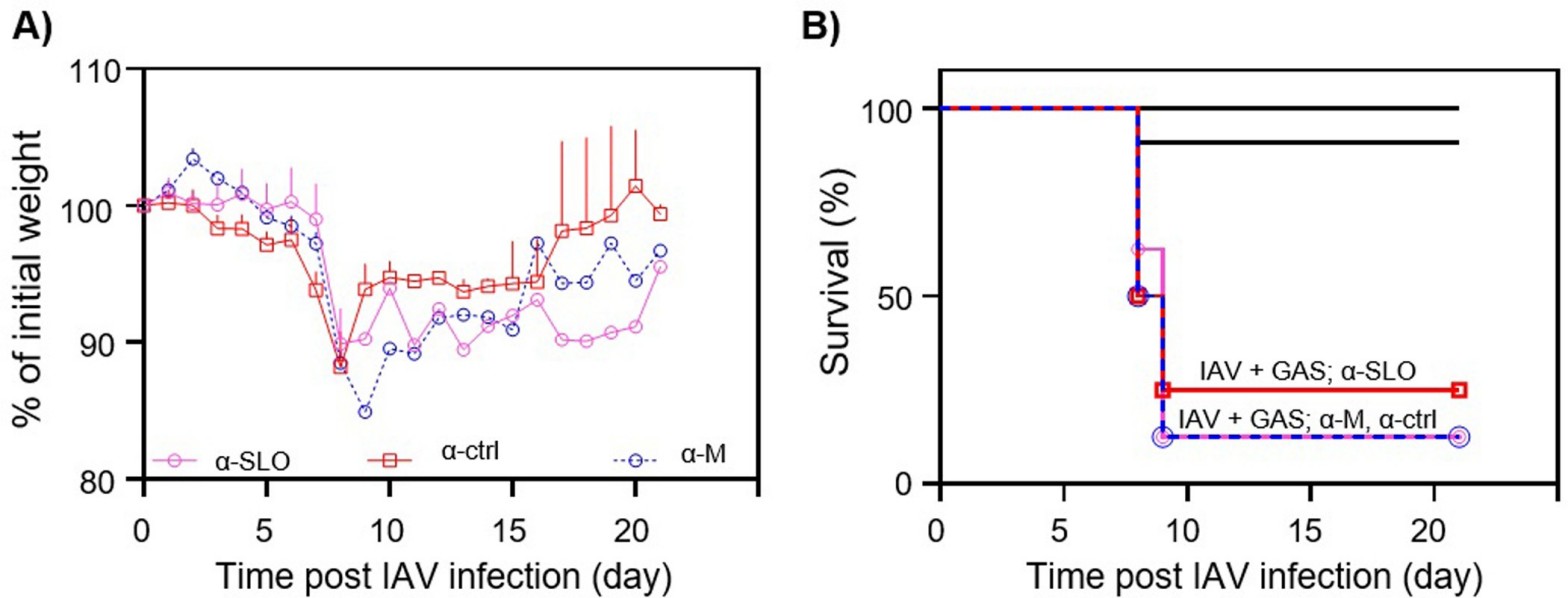
Determination of the effect of therapeutic administration of antisera to SLO (α-SLO) or the M protein (α-M) on morbidity (weight loss) and mortality of IAV-GAS superinfected mice. Mice were injected with antibodies specific to SLO (α-SLO) or the M protein (α-M) 2 h after superinfection with GAS. For controls, groups of mice were treated with non-immune sera (α-ctrl). Treatment with α-SLO decreased morbidity compared to treatment with non-immune sera (*p* < 0.001). There were no differences in mortality between treated and untreated groups of mice (*p* > 0.05). The area under the curve analysis (days 8–15) was completed prior to an unpaired Student’s *t*-test to compare the differences in morbidity. A Kaplan–Meier survival analysis was used to compare the differences in mortality between the groups.

To determine if treatment with antisera to SLO or the M protein could decrease the morbidity or mortality associated with an established IAV-GAS superinfection, mice were first inoculated with a sublethal dose of IAV (0.1 LD_50_) on day 0. On day 7 mice were inoculated with a sublethal dose of GAS (0.1 LD_50_) and 2 h later were given antisera to either SLO or the M protein. As a control, mice were similarly treated with non-immune sera. Treatment with antisera to the M protein did not decrease morbidity compared to mice receiving non-immune sera (*p* > 0.05; Fig 6A). Treatment with antisera to SLO decreased morbidity compared to mice treated with non-immune sera (*p* < 0.001; Fig 6A); however, treatment with neither antisera significantly reduced mortality compared mice treated with non-immune sera (*p* > 0.05; Fig 6B).

## Discussion

Despite the availability of influenza vaccines, anti-viral agents, and antibiotics that are effective against GAS *ex vivo*, the morbidity and mortality of IAV-GAS superinfections is considerable. We assessed the use of passive immunotherapy to reduce morbidity and mortality using an established murine model of IAV-GAS superinfection and antisera targeting two GAS proteins; one against the cell wall-associated M protein and the other against the exoprotein SLO. We measured efficacy with three endpoints: i) enumeration of GAS in the blood and organs of mice. ii) weight loss (morbidity). iii) mortality. In general, prophylactic use of the antisera decreased morbidity compared to groups of mice treated with non-immune sera. Therapeutic treatment with the antisera slightly decreased the numbers of bacteria in the lungs and spleens of superinfected mice but there was no significant decrease in mortality. While refinement of the antibody formulation is necessary, our results support the continued development of passive immunotherapy as a potential preventative measure, or treatment, for IAV-GAS superinfections.

Both the M protein and SLO enhance virulence by decreasing the susceptibility of GAS to killing by immune effector cells such as macrophages and neutrophils [36–40]. Passive antibody treatment can decrease disease by two or more mechanisms. First, antibodies can promote opsonophagocytosis, which is consistent with our interpretation of results assessing the survival of GAS incubated with antibodies to the M protein and suspended in whole blood (Fig 2). In this regard, serotype-specific opsonic antibodies to the cell wall localized M protein have been known for decades to be important in the clearance of GAS and are typically protective [41]. In contrast, SLO is an exoprotein that disrupts the integrity of cholesterol-containing cell membranes [36], which can induce caspase-dependent apoptosis in neutrophils, macrophages [37], and epithelial cells [38]. Antibody-mediated neutralization of SLO [42] can decrease toxin-mediated apoptosis of immune effector cells and thereby increase the killing of GAS. Similarly, antibodies to the exoprotein streptolysin S enhanced GAS killing by neutralizing the cytolytic activity of the toxin [43]. Finally, active vaccination of non-human primates with an SLO toxoid (also containing other GAS antigens) induced non-opsonizing SLO-specific antibodies and decreased pharyngitis [44]. Thus, the antisera to the M protein and SLO likely enhanced opsonophagocytosis (Fig 2) and neutralized cytolytic activity (Table 1), respectively.

Our study was not designed to compare the efficacy of antisera against the M protein to that against SLO but rather to assess the feasibility of pursuing such an approach. We note that treatment was not based on the titer of antigen-specific antibodies, which differed between the sera.

The mortality of iGAS diseases is remarkably high despite the susceptibility of the pathogen to antibiotics and there is considerable need for adjunct therapeutics. Patients with iGAS diseases typically have either low titers, or lack antibodies to the causative GAS serotype suggesting that sufficient levels of circulating antibodies may protect against iGAS diseases [45, 46]. Intravenous immunoglobulin (IVIG) contains both opsonic and neutralizing antibodies to several GAS virulence factors [47–52]. There has been some clinical success using IVIG to treat iGAS diseases, especially streptococcal toxic shock syndrome [51, 53, 54]; however, definitive results from robust clinical trials are lacking [55, 56]. This is due, in part, to challenges in enrolling a sufficient number of patients and probably the low concentration, and variability, of GAS-specific antibodies in IVIG [57]. A study supporting this interpretation found that when IgG antibodies specific to GAS were purified from human IVIG they were more efficacious when used as a passive vaccine in a murine model of iGAS infection compared to IVIG [58]. These results support the idea that GAS-specific antibodies may be an effective therapeutic approach to manage iGAS diseases, although it is acknowledged that IVIG treatment also dampens the inflammatory response [59], which likely affects clinical outcomes.

Passive antibody therapy has been successfully used to treat toxin-mediated bacterial diseases including diphtheria, tetanus, botulism, and *Clostridium difficile* infections [60]. These diseases result largely from the activity of one, or two, specific proteins. In contrast, the pathogenesis of GAS (and many other bacteria), involves multiple, sometimes functionally redundant, virulence factors. Thus, the successful development of antibody-based adjunct therapeutics to pathogens employing multiple virulence factors may require formulations that include antibodies to multiple virulence factors and not monoclonal or polyclonal antibodies targeting a single protein. Subsequent studies will explore the efficacy of using a combination of IgG antibodies to both the M protein and SLO in mitigating the morbidity and mortality of IAV-GAS superinfections.

Several studies have used animal models to test the ability of passive immunotherapy to protect against iGAS diseases, although ours is the first to assess efficacy in the context of a viral superinfection. Previous studies showed that opsonic antisera to a fibronectin binding protein (FBP54) [61], the streptococcal hemoprotein receptor (Shr) [62], GAS carbohydrate [63], or various epitopes of the M protein [64–66] are protective compared to controls. In addition, passive immunization with non-opsonic antisera containing antibodies to Streptococcal esterase (Sse) [67], streptococcal pyrogenic exotoxin A (SpeA) [68], the M6 protein [69], or C5a peptidase [70] are also protective based on studies using mice. Taken together, the studies show that the prophylactic use of either opsonizing or non-opsonizing antibodies targeting GAS can be useful in the protecting against a GAS monoinfection; however, there has been less success in treating established iGAS infections using passive immunotherapy.

While an active vaccine against GAS is an ideal outcome, many successful vaccines do not abolish disease including the pneumococcal, Hib, and influenza vaccines [71–74]. Therefore, the development of therapeutic antibodies designed as an adjunct treatment for severe iGAS diseases, including IAV-GAS superinfections, may prove to be beneficial, particularly during influenza pandemics.
